# Altering the Elastic Properties of 3D Printed Poly-Lactic Acid (PLA) Parts by Compressive Cyclic Loading

**DOI:** 10.3390/ma13194456

**Published:** 2020-10-08

**Authors:** Tomaž Pepelnjak, Ako Karimi, Andraž Maček, Nikolaj Mole

**Affiliations:** 1 Faculty of Mechanical Engineering, University of Ljubljana, Aškerčeva 6, SI-1000 Ljubljana, Slovenia; tomaz.pepelnjak@fs.uni-lj.si (T.P.); andraz.macek@fs.uni-lj.si (A.M.); 2 Kolektor Etra d.o.o., Šlandrova ulica 10, SI-1231 Ljubljana—Črnuče, Slovenia; ako.karimi@kolektor.com

**Keywords:** 3D printing, FFF, PLA, infill line distance, compression test, loading–unloading

## Abstract

In designing high-performance, lightweight components, cellular structures are one of the approaches to be considered. The present study aimed to analyze the effect of the infill line distance of 3D printed circular samples on their compressive elastic behavior. Lightweight cellular poly-lactic acid (PLA) samples with a triangular infill pattern were exposed to cyclic compressive loading and their stiffness was investigated. PLA is one of the most commonly used thermoplastic materials in additive manufacturing using the fused filament fabrication (FFF) process. Cylindrical samples with a diameter of 11.42 mm and a height of 10 mm were printed using FFF technology with two different infill line distances (1.6 mm and 2.4 mm). Comparing the nominal compressive stress-nominal strain curves under cyclic loading showed that the first cycle response was significantly different with respect to the subsequent ones. Furthermore, an analysis of the dependence of the modulus of elasticity on the effects of cyclic loading was performed. It was found that through elastic deformation, and combined elastic and plastic deformation, the samples’ properties such as stiffness could be altered.

## 1. Introduction

Additive manufacturing (AM) methods, and 3D printing, in particular, are highly important for industry. 3D printing is a typical example of an AM technology and one that has gained in popularity over the previous decade due to the reduction in the costs of printing equipment and materials as well as its broad applicability and implementation into Industry 4.0 [1]. Material extrusion is the most widespread AM technology [2]. There is an increasing demand for knowledge on how to properly use and benefit from fused filament fabrication (FFF) technology. This technology, as part of the AM concept, has become very popular in recent years due to the increasing availability of low-cost small and portable 3D printers. In addition to reducing waste, this method also enables the fabrication of parts with complex geometries while requiring little supervision and protection during production [3,4,5]. Through the efforts of the scientific and research community, properties such as the strength of materials used in 3D printing have been improved [6,7,8,9].

Thermoplastic polymer materials most commonly used for extrusion in additive manufacturing by the FFF method are poly-lactic acid (PLA) and acrylonitrile butadiene styrene (ABS). According to many researchers, PLA can be a suitable replacement for ABS plastic, which is toxic; for this reason, the use of PLA in desktop 3D printing is increasing. PLA is a biodegradable polymer and is currently used in packaging and for the production of disposable parts; studies are being conducted on these parts in order to develop improvements that would allow the use of PLA in durable applications [10,11,12,13,14,15,16]. In this article, the mechanical behavior of PLA parts produced by FFF technology is investigated.

Studies and research conducted over the past few years on the mechanical properties of products manufactured by 3D printers have aimed to enable the production of better and more capable products. The results show that the products printed by home desktop 3D printers (e.g., the RepRap 3D printer with low cost and small size that can be used at home, universities, etc.) using the FFF method can exhibit tensile strength equal to that of the products produced by commercial printers [17]. Due to the interest shown by the scientific community and the attractiveness of the subject, some recent publications have focused on the mechanical characteristics of the produced parts using FFF technology. These papers include one by Goh et al. [18], which investigated the sustainability of thermoplastics; one by Chacon et al. [19], which investigated the effects of the production parameters on the mechanical properties of PLA parts; and another by Tymrak et al. [17], which focused on ABS and PLA. The raster angle of 3D printing, the width and height of printed layers, and the orientation of the part during printing are the factors identified by Forster [20] as parameters that affect the mechanical properties. 

Material extrusion is one of the types of AM technology that have been investigated by different researchers and authors [21]. In this paper, we concentrated on the FFF process. Due to the thickness of the layers (0.1–0.3 mm) in the FFF method in comparison to the thickness of the layers in other 3D printing technologies such as stereo-lithography, which has a 50 to 100 μm layer height, the parts created by the FFF method have a rougher surface. Wittbrodt et al. [22] studied the crystallinity and tensile strength of such samples. 

In a paper by Carrasco et al. [23], the tensile yield strength and the processing temperature at injection molding and extrusion/injection of PLA were observed and compared with other polymeric materials. By evaluating the physical properties of PLA in comparison with commodity polymers, we can observe that the tensile yield strength for PLA, polystyrene (PS), and polyethylene terephthalate (PET) is 48–110 MPa, 34–46 MPa, and 47 MPa, respectively. The processing temperatures for PLA, PS, and PET are 210 °C, 230 °C, and 255 °C, respectively. Young’s modulus of PLA is likewise higher in comparison with PS and PET [24,25,26], ranging from 3.5 to 3.8 GPa. 

The effects of printing parameters on the mechanical properties of 3D-printed parts using the FFF method were investigated by Ćwikła et al. [27]. Their experiments investigated infill density, infill pattern, solid layers, and the extrusion multiplier at constant printing speed. Extrusion and print bed temperatures were 240 °C and 100 °C, respectively. The tensile tests were performed at room temperature (22 °C) and at a humidity of 50%, and the strain rate was 10 mm/min. The authors found that an increase in density (increased infill value) reduced deformation. The strength of the samples increased with the increase in infill density. The authors also investigated the influence of shell thickness (encompassing the perimeter in the horizontal plane and the solid layers forming the bottom and the top) and found that the shell thickness (i.e., the number of layers) had a large influence on the tensile strength of the samples.

Rodríguez-Panes et al. [28] analyzed the mechanical behavior of parts produced using FFF technology by comparing PLA and ABS thermoplastic materials. The tensile yield stress *R*_p_, tensile strength *R*_m_, nominal strain, and Young’s modulus were investigated. The PLA samples had higher tensile strength than those from ABS. The adhesion between the layers, which was higher in PLA materials, makes these very suitable for use in AM.

The possibility of increasing the strength of samples printed by a 3D Ultimaker 2 printer through optimizing the parameters of the FFF process was studied by Kuznetsov et al. [29]. Three different modifications were made to the FFF process: the printer cooling was deactivated, the extrusion temperature was increased, and the thickness of the printed layers was decreased. They concluded that a 108% increase in the strength of the printed samples was possible by adjusting the printing parameters. It can generally be said that part strength can be optimized by fine-tuning the printing process parameters.

Singh Mehta et al. [30] performed compression tests of PLA samples produced by 3D printing technology to analyze their mechanical properties. The results showed that by increasing infill density, shell thickness, and layer height, the compression strength of PLA materials was also increased. Similarly, Mercado-Colmenero et al. [31] presented a numerical and experimental study on the compression properties of PLA materials produced by FFF technology. Tahseen et al. [32] investigated the effect of infill density on the compression properties of PLA samples produced by FFF technology. The results of the study suggest that to achieve high mechanical strength, high infill density should be selected.

In the research of Liang et al. [33], cylindrical samples with different structures such as tetragonal, hexagonal, and wheel-like were designed and manufactured from PLA material by 3D printers using FFF technology. The compressive properties of these samples, both experimentally and theoretically determined, were studied and compared, and the fatigue behavior of the samples under cycling loading was also analyzed. By examining the stress–strain curves of the samples produced with three different structures after the pressure test, it was found that the sample with the tetragonal structure had the highest compression modulus. This infill shape was, therefore, also selected for the studies of cyclic loading of FFF printed specimens in the present paper.

An overview of printing speeds, printing temperatures, and layer heights used by different researchers can be found in Table 1.

Based on the data presented in Table 1, we followed the above recommendations, choosing the printing speed and printing temperature in the same range.

The major goal of our work was to improve the mechanical properties without increasing the weight of the structure; we wanted to show that it is possible to improve the mechanical properties of the polymer structure produced by additive manufacturing by additional elastic and/or plastic deformation. Deliberately deforming polymer structures is a novel approach, not yet found in the scientific literature, and it is expected to aid in engineering lightweight structures. In this paper, the mechanical properties were altered through elastic deformation as well as combined elastic and plastic deformation of the samples. Furthermore, the influence of the major technological parameters of the FFF process on the changes induced in the material by cyclic loading were analyzed.

## 2. Equipment and Materials 

### 2.1. 3D Printer

The 3D printer used in our research to produce the FFF printed samples was an Ultimaker 3 by Ultimaker BV, Geldermalsen, Netherlands [36] with a nozzle diameter of 0.4 mm.

### 2.2. Optical Microscope

The structure of the printed samples was analyzed using the optical microscope Keyence VHX–6000, Osaka, Japan, with adaptive multi-lightning, advanced auto-focusing, and high-definition imaging in one system in order to provide maximum image resolution. Using various VH-series lenses, it enables magnification ranging from 0.5× to 5000×. Other technical characteristics of the optical microscope used in the present study were that the camera had 18 megapixels and a 1/18-inch CMOS image sensor.

### 2.3. Mechanical Testing Machine

**Figure 1:**
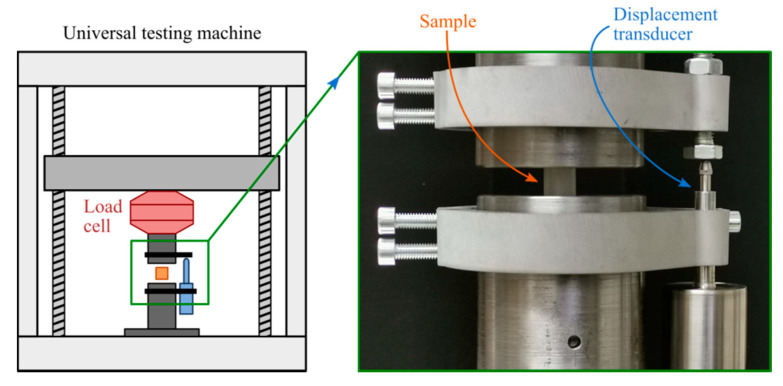
Schematic diagram of the UTM (**left**) and the sample located between the compression plates (**right**).

A universal testing machine (UTM) FS-LNMS was used to perform the compression tests of the analyzed samples (Figure 1, left). This UTM has a capacity of 30 kN and is driven by a servo motor. Flat compression plates were used to grip the specimens (Figure 1, right). The load cell for force acquisition was AST KAF-W, Gruppe Angewandte System Technik GmbH, Dresden, Germany, with a maximum force of 1 kN and an accuracy class of ± 0.05%. Information about the linear variable differential transducer (LVDT) used is presented in Table 2, while Table 3 shows the main compression testing parameters.

Cyclic compressive loading tests were performed on PLA samples produced by 3D printing technology with infill line distances (ILD) of 2.4 mm and 1.6 mm, focusing on the mechanical properties. Our tests were performed to validate the experimental tests on six samples with ILD of 2.4 mm and six samples with an ILD of 1.6 mm, all of which had similar geometric and manufacturing characteristics. The samples were located between the surfaces of the compression plates so that the end surfaces of the samples were parallel to the surfaces of the compression plates. The diagrams of the uniaxial compression force in relation to nominal compression displacement were acquired. 

### 2.4. PLA Material

PLA is a thermoplastic produced from natural sources such as corn and is the most popular material used in 3D printers due to its relatively low melting point and its low environmental impact. This material requires relatively small amounts of energy to be printed by 3D printers, since it has a melting point between 180 °C and 220 °C, and a glass transition temperature between 60 and 65 °C [37,38]. The glass transition temperature (*T*_g_) of the PLA material leads to good adhesion of the printed layers, and it is also suitable for maintaining the shape of the material at medium operating temperatures.

In this article, PLA filament with a diameter of 2.85 mm and a density of 1.24 g/cm^3^ was used to produce samples. The properties of the PLA filament according to the manufacturer, are as follows:

- Material (filament manufacturer): Ingeo Biopolymer 4043D,
- Color: transparent,
- Diameter: 2.85 mm,
- Density: 1.24 g/cm^3^,
- Printing temperature: from 190 °C to 230 °C,
- Glass transition temperature: from 55 °C to 60 °C,
- Tensile yield strength: 60 MPa,
- Young’s modulus: 3600 MPa,
- Flexural strength: 83 MPa,
- Flexural modulus: 3800 MPa.

### 2.5. Fused Filament Fabrication (FFF) Parameters

**Figure 2:**
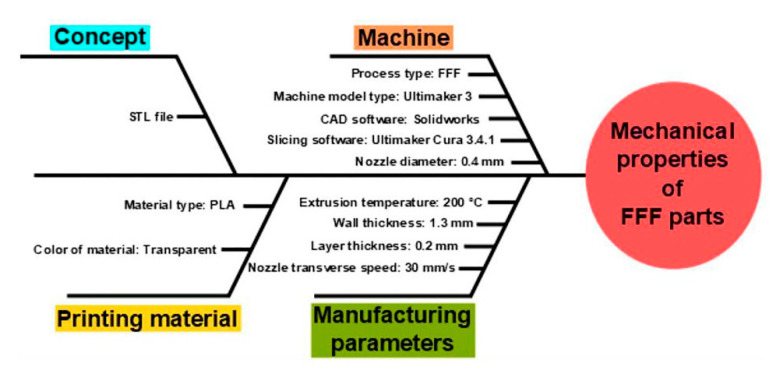
Summary of our research parameters that have a role on the resulting mechanical properties of FFF technology in Ishikawa diagram.

The two main groups of FFF parameters are the (a) manufacturing and (b) structural parameters. The printing speed or deposition rate, the extrusion temperature, the temperature of the build platform, the ambient temperature, and similar factors can be included in the (a) group. The (b) group includes, among others, the gap between layers, the raster orientation, and the orientation of the printed part, as described by Cuan-Urquizo [39]. The fishbone chart in Figure 2 shows a summary of our selected printing parameters.

### 2.6. Preparation of the Samples

The samples in the form of an open-top cylinder of D = 11.42 mm (diameter) and H = 10 mm (height) were designed using Solidworks software and saved as STL files. 

In the next step, we used Cura 3.4.1 software and started the process of converting the 3D model to a print file as described below:

- Load a 3D model (STL file) into the Cura software.
- Select the Ultimaker 3 printer from the list of printers.
- Select PLA material from the list of materials.
- Adjust the print settings as follows:
  - Layer height: 0.2 mm,
  - Infill pattern: triangles,
  - Infill line distance: 1.6 mm (samples named “A”), 2.4 mm (samples named “B”),
  - Printing temperature: 200 °C,
  - Print speed: 30 mm/s,
  - Wall thickness: 1.3 mm.
- With the settings adjusted and the samples placed on the print bed in the right position in Cura 3.4.1 software, the model is ready to print. All that needs to be done is to save the 3D print file as a G-code file and send it directly to the Ultimaker 3 printer for printing.

**Figure 3:**
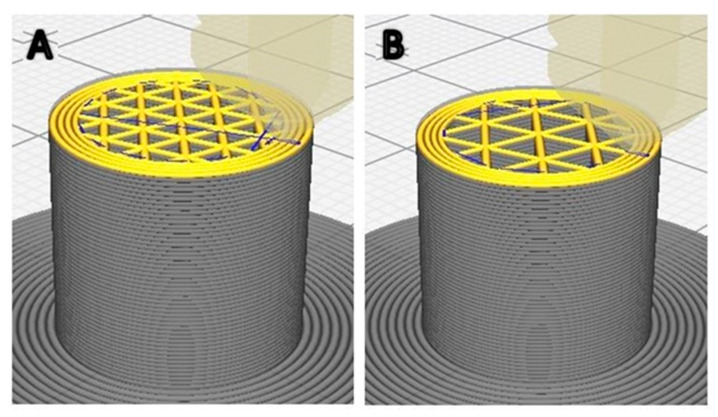
3D modeling images of the 3D-printed samples (from Cura 3.4.1 software) with different infill line distance designed in this study. (**A**) 1.6 mm ILD, (**B**) 2.4 mm ILD.

In Figure 3, the 3D model of the 3D-printed samples tested in this article can be seen in the Cura 3.4.1 software environment. From Figure 3, it is evident that the wall structure is produced in four circles, while the infill pattern is produced with only one PLA wire. The real width of the printed wire is not evaluated in the Cura environment, and the produced parts reveal the overlapping of the wall layers. 

#### 2.6.1. Calculation of Real Infill Density in Samples with Different ILD

**Figure 4:**
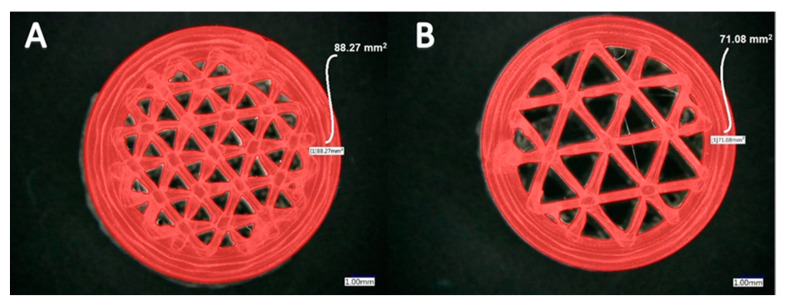
Area measurement of infill lines: (**A**) 1.6 mm ILD, (**B**) 2.4 mm ILD.

**Figure 5:**
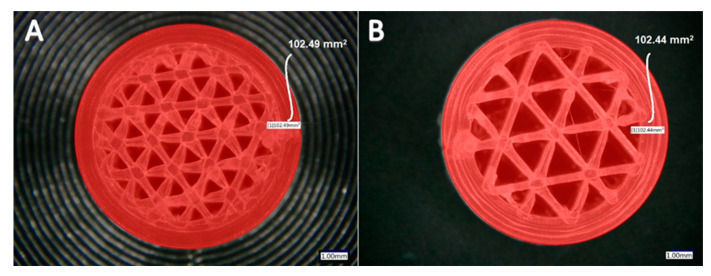
Area measurement of fill surface: (**A**) 1.6 mm ILD, (**B**) 2.4 mm ILD.

In Cura software, some options such as infill density and ILD are excluded and affect each other. It is possible to set the infill line distance instead of setting the infill density as a percentage, which determines the distance between two infill lines, which has the same effect as changing the infill density. This is why we decided to focus on the ILD option, choosing it as the main property to investigate in our research. We started the process of measuring infill density for samples with 1.6 mm and 2.4 mm ILD. The steps for calculating the area of infill structure (Figure 4 and Figure 5) using a microscope are as follows:

- After the sample is placed under the microscope, the area of measurement is chosen.
- The user selects the desired area manually using the drawing tools available in the microscope software, which automatically delivers the total of the user-acquired area. The area of the sample cross-section of the software model is also measured. In this research, the area of the sample cross-section being measured from the model in CAD software is called the ideal area of the sample cross section.

Calculated infill density of 1.6 mm ILD samples:

- Measured area of the sample cross-section: 88.27 mm² (Figure 4A)
- Ideal area of the sample cross-section (software): 78.27 mm²
- Measured area / Ideal area = 88.27 mm² / 78.27 mm² = 1.12
- Measured area of the cross-section / Measured area of fill cross-section =
- 88.27 mm² / 102.49 mm² = 0.86
- Infill density ≅ 86%

Calculated infill density of 2.4 mm ILD samples:

- Measured area of the sample cross-section: 71.08 mm² (Figure 4B)
- Ideal area of the sample cross-section (software): 66.74 mm²
- Measured area / Ideal area = 71.08 mm² / 66.74 mm² = 1.06
- Measured area of the cross-section / Measured area of fill cross-section = 71.08 mm²/102.44 mm² = 0.69
- Infill density ≅ 69%

**Figure 6:**
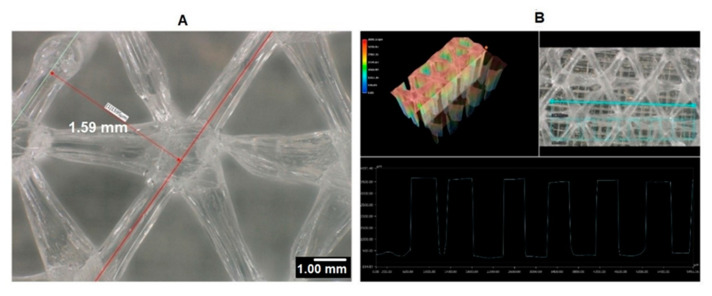
(**A**) Microscopic picture from top view of the 3D-printed sample with 1.6 mm ILD at 100× magnification setting. (**B**) 3D profile of the 3D-printed sample with 1.6 mm ILD at the 100× magnification setting.

**Figure 7:**
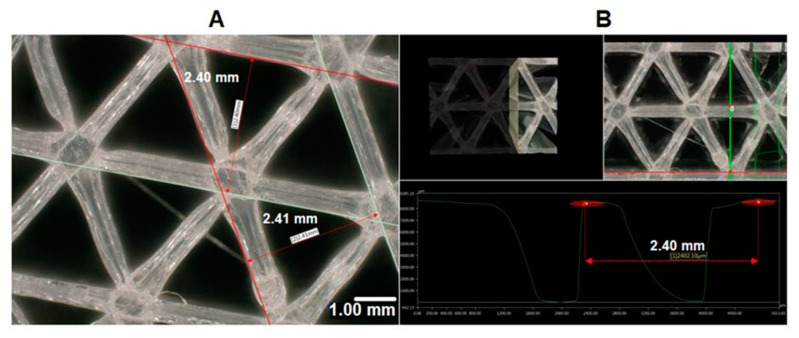
(**A**) Microscopic picture from top view of the 3D-printed sample with 2.4 mm ILD at the 100× magnification setting. (**B**) 3D profile of the 3D-printed sample with 2.4 mm ILD at the 100× magnification setting.

For a better presentation of ILD on the real samples, 3D scanning was performed; the results are presented in Figure 6 and Figure 7.

#### 2.6.2. Nozzle Movement Path Analysis

**Figure 8:**
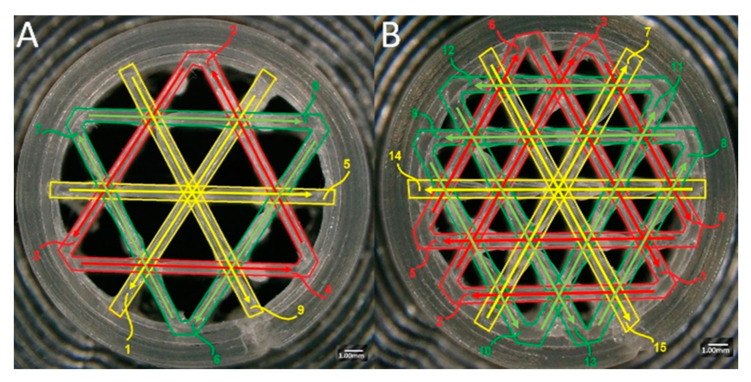
The movement path of the printer nozzle when printing: (**A**) 2.4 mm ILD sample, (**B**) 1.6 mm ILD sample.

The infill structure was produced with linear movements of the printer nozzle when printing with 2.4 mm and 1.6 mm ILD. The movement sequences are shown with numbers 1 to 9 for the 2.4 mm ILD and 1 to 15 for the 1.6 ILD (Figure 8).

Microscopic examination of the samples revealed that the shape quality of the infill lines is related to the movement path of the nozzle. Following analysis of individual layer, the shape quality of the first line printed was seen to be better than the shape quality of the second line printed; similarly, the shape quality of the second line was better than that of the third one, etc. In general, the first straight-line path had the best shape quality, and the last straight-line path (the last infill line printed by the printer) had the worst shape quality.

#### 2.6.3. Cutting the PLA Samples and Obtaining the Cross-Section for Microscopic Analysis

##### Low Speed Cutting Machine

We tested cutting with knives, cryogenic cutting with knives, and cutting with microtome knives for obtaining the cross-sections of the 1.6 mm and 2.4 mm ILD samples. The simplest and most accessible method for cutting PLA samples for the preparation of it for microscopic analysis in our research was the use of a low-speed sawing machine. The machine used in our research to cut the samples was a Buehler IsoMet^TM^, Illinois Tool Works, Lake Bluff, Illinois, USA, low-speed saw [40]. This is a precision sectioning saw that was designed to achieve acceptable cut surface quality for delicate samples at low speeds (0–300 rpm). It uses gravity to provide the cutting force to reduce the deformation of delicate samples and features a precision micrometer for specimen alignment. Further details are as follows:

- Type of blade: diamond wafering blades (4 in blade),
- Speed range: 0–300 rpm,
- Component positioning accuracy: ±5 μm positioning via a manual micrometer.

##### Sample Cross-Section for Microscopic Analysis

**Figure 9:**
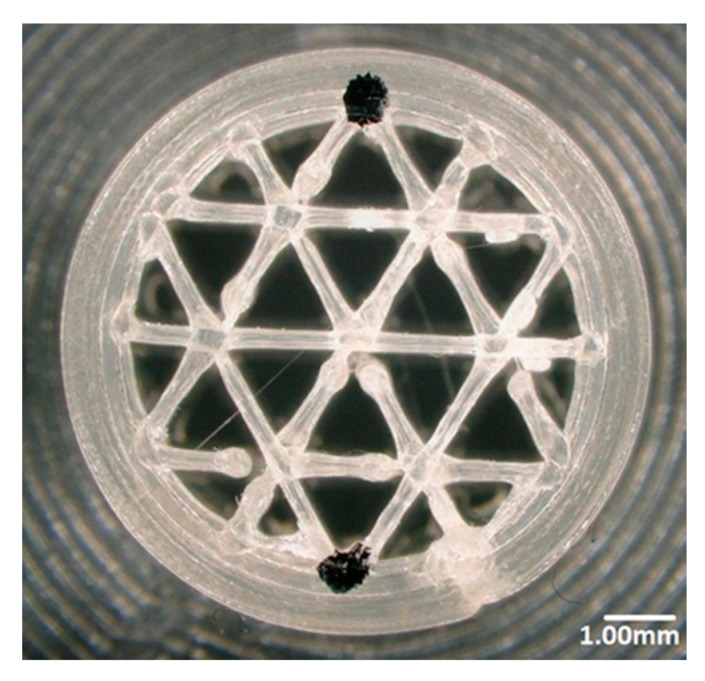
Two points were marked on the up surface of the samples.

**Figure 10:**
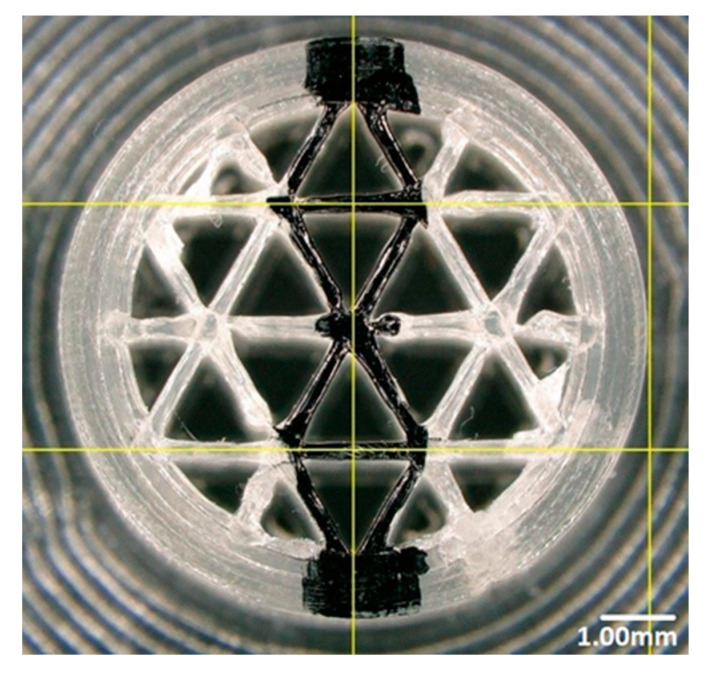
The marked points were connected together under the microscope.

The steps for preparing the sample cross-section for microscopic analysis are detailed below:

- The samples (1.6 mm and 2.4 mm ILD) were placed one by one under a microscope; on the top surface of each sample, two points were marked using a marker (see Figure 9). With a microscope, we attempted to locate the points in all samples, with the least amount of error, crossing over the best quality lines.

- After marking the samples and specifying the two points on each of them, the marked points were connected using a marker and a ruler under the microscope to improve the positioning of the sample on the cutting machine (Figure 10).

- The samples with lines drawn on were placed onto the low-speed cutting machine in such a way that the blade of the machine was perpendicular to the surface (marked with the line) of the samples. Using the manual micrometer of the cutting machine, the position of the blade was adjusted exactly at the location of the drawn line.

**Figure 11:**
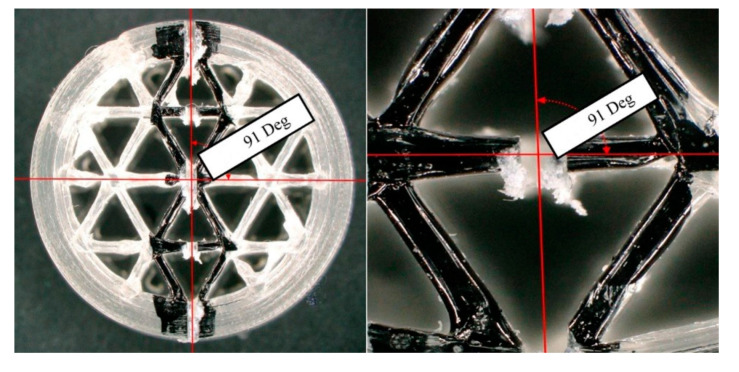
The accuracy of the cuts made on the 2.4 mm ILD sample.

Figure 11 shows the accuracy of the cuts made on the 2.4 mm ILD sample.

**Figure 12:**
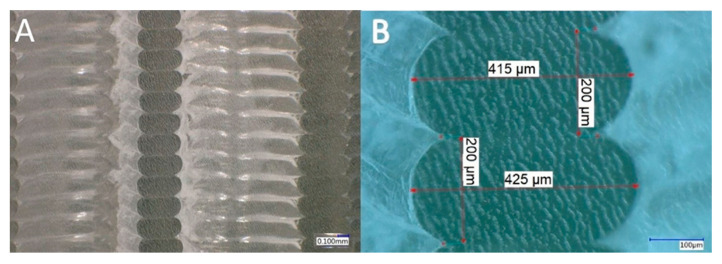
Microscopic cross-section image of 3D-printed sample with 1.6 ILD before compression test (cyclic loading–unloading). (**A**) 100× magnification setting, (**B**) 500× magnification setting.

**Figure 13:**
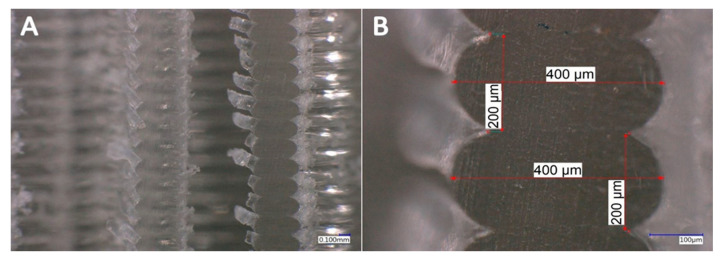
Microscopic cross-section image of 3D-printed sample with 2.4 ILD before compression test (cyclic loading–unloading). (**A**) 100× magnification setting, (**B**) 500× magnification setting.

In Figure 12 and Figure 13, the microscopic cross-section image of the 3D-printed sample with the 1.6 mm and the 2.4 mm ILD before the compression test can be seen at the different magnification settings. Since the perpendicularity of the cut also influences the observed shape of the printed material on the cross-section, the accuracy of the positioning was analyzed with preliminary tests. The angular error was less than one angular degree. The measured cross-sections of the unloaded samples showed a layer height of exactly 0.2 mm, while the measured width ranged from 0.400 mm to 0.425 mm.

**Figure 14:**
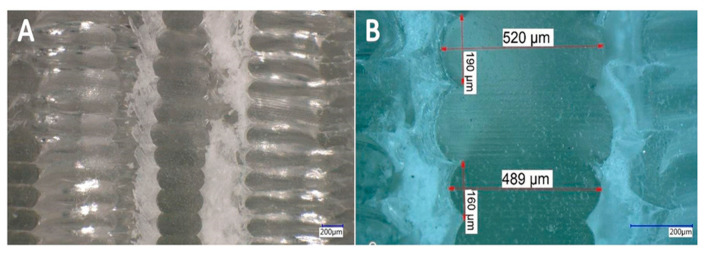
Microscopic cross-section image of 3D-printed sample with 1.6 ILD after compression test (cyclic loading–unloading). (**A**) 100× magnification setting, (**B**) 300× magnification setting.

**Figure 15:**
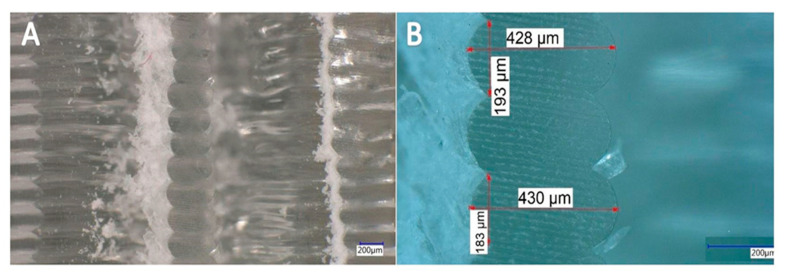
Microscopic cross-section image of 3D-printed sample with 2.4 ILD after compression test (cyclic loading–unloading). (**A**) 100× magnification setting, (**B**) 300× magnification setting.

After performing the compression test (loading–unloading cycles) on the samples, the microscopic images of the cross-sectional area can be observed at different magnifications in Figure 14 and Figure 15. 

By comparing the microscopic images of the cross-sectional area of the 1.6 mm and the 2.4 mm ILD samples before and after performing the compression test, it can be seen that some layers of the samples had deformed and the shape of the cross-sectional area of some layers had irreversibly changed due to the cyclic loading. It is possible to customize the part properties by targeted application of plastic deformation, and it is possible to improve mechanical properties like hardness by additional loading.

These images show that irreversible deformation occurred after the samples were exposed to loading–unloading. Microscopic images (after loading) belong to the second mode, in which samples were cyclically loaded from the initial displacement value of 0.4 mm to 0.5 mm.

## 3. Results and Discussion

### The Influence of Infill Line Distance on the Compressive Strength of PLA

In our research, two types of samples were printed and fabricated; other than the different infill line distance, we used the same print settings.

**Figure 16:**
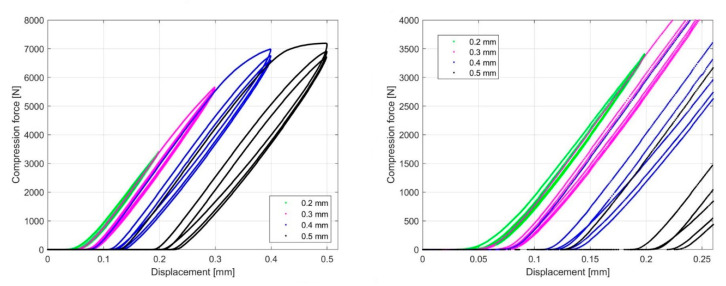
Compression force–displacement diagram for 1.6 mm ILD.

**Figure 17:**
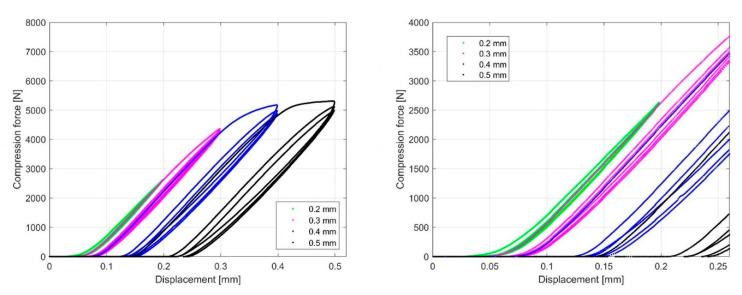
Compression force–displacement diagram for 2.4 mm ILD.

The compression test was performed the following way: each sample was cyclically loaded to a nominal displacement value that was increased every 10 cycles by a step of 0.1 mm in the interval from 0.2 mm to 0.5 mm. The initial nominal displacement values are different from sample to sample and start at 0.2 mm and 0.4 mm. In Figure 16 and Figure 17, the force–displacement curves obtained by cyclic loading for the 1.6 mm and 2.4 mm infill line distance are presented. Comparing the curves under cyclic loading at individual nominal displacement values showed that the first cycle response was significantly different with respect to the subsequent cycles. Irreversible deformation was evident in the first cycle when the nominal displacement values were 0.4 mm and 0.5 mm. The hysteresis loop represents the mechanism of cyclic hardening within the samples. The hysteresis decreases from cycle to cycle at a given nominal displacement.

**Figure 18:**
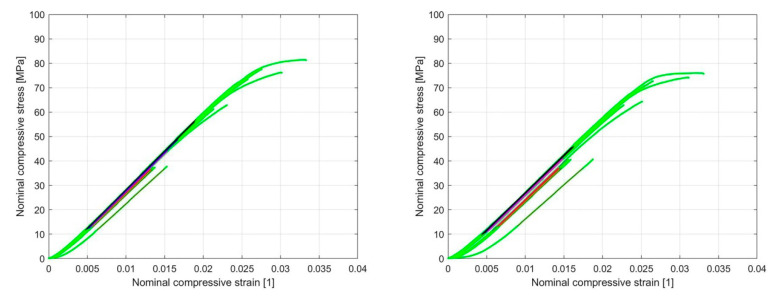
Nominal compressive stress–nominal compressive strain diagram with the linear region of the curve highlighted for 1.6 mm (**left**) and 2.4 mm (**right**) ILD (initial nominal displacement 0.2 mm).

**Figure 19:**
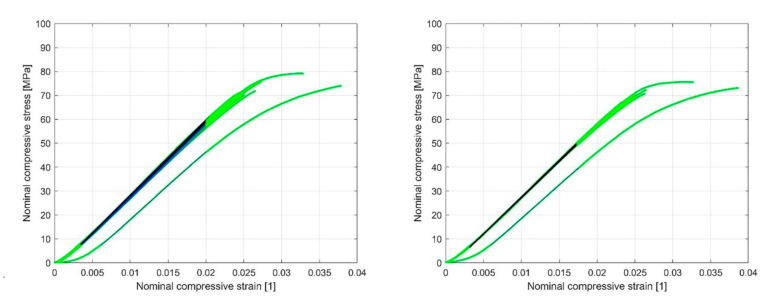
Nominal compressive stress–nominal compressive strain diagram with the linear region of the curve highlighted for 1.6 mm (**left**) and 2.4 mm (**right**) ILD (initial nominal displacement 0.4 mm).

**Figure 20:**
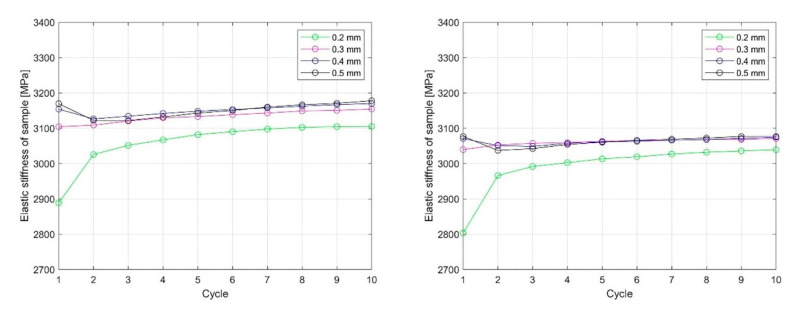
The dependency of the ES of the sample on the number of cycles and the loading path for 1.6 mm (**left**) and 2.4 mm (**right**) ILD (initial nominal displacement 0.2 mm).

**Figure 21:**
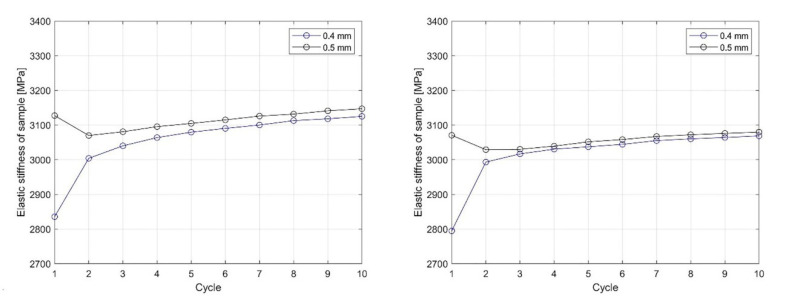
The dependency of the ES of the sample on the number of cycles and the loading path for 1.6 mm (**left**) and 2.4 mm (**right**) ILD (initial nominal displacement 0.4 mm).

Based on the linear region identified on the nominal compression stress–nominal strain curve (Figure 18 and Figure 19), which determines the linear elastic response of the sample, an analysis of the influence of cyclic loading on the elastic stiffness (ES) of the sample was performed (Figure 20 and Figure 21). This analysis was made for two cases. 

In the first case, the sample was cyclically loaded from an initial nominal displacement value of 0.2 mm up to a displacement of 0.5 mm in steps of 0.1 mm. At nominal displacement values of 0.2 mm and 0.3 mm, no irreversible deformations were observed, while at values of 0.4 mm and 0.5 mm, irreversible deformations occurred only in the first cycle. 

In the second case, the sample was cyclically loaded from an initial displacement value of 0.4 mm to 0.5 mm. Here, too, 10 cycles were performed at each nominal displacement value. At each nominal displacement value, irreversible deformations were only observed in the first loading cycle.

The nominal compression stress $\sigma_{c}$ is defined as

(1) $$\sigma_{c}=\frac{F}{\;A_{0}\;}$$

where *F* is the resultant compression force on the sample and $A_{0}$ is the initial cross-section area of the sample before loading, determined experimentally for each specimen as described in Section 2.6.1. The nominal compressive strain $\varepsilon_{c}$ is computed with the following equation

(2) $$\varepsilon_{c}=\frac{\Delta H}{H_{0}}$$

In Equation (2), ΔH is the sample height change due to the compressive load and $H_{0}$ is the initial height of the sample.

To obtain the ES of the sample, the polyfit MATLAB function was used on a set of data points defined by the nominal compression strain and nominal compression stress to fit the linear curve to the obtained dataset. The slope of the obtained linear part of the curve for the individual load cycles (Figure 18 and Figure 19) represents the ES of the sample. In Figure 20 and Figure 21, the cycle dependence of the ES of the sample is shown for the two infill line densities that were analyzed. Figure 20 and Figure 21 show that the ES of the sample increased during cyclic loading, with most of the increase occurring at the initial nominal displacement (i.e., at values of 0.2 mm (interval [0.2 mm, …, 0.5 mm]) and 0.4 mm (interval [0.4 mm, 0.5 mm])). At a nominal displacement of 0.4 mm and 0.5 mm, inelastic deformation was observed only in the first cycle. After the first cycle, the ES of the sample decreased. The next nine loading cycles were in the elastic domain of deformation and the ES of the sample slowly increased again. Comparing the response for 1.6 mm and 2.4 mm ILD, we could determine the difference in the ES of the sample, which was 3% lower in the case of 2.4 mm ILD than in the case of 1.6 mm ILD.

With compression loading, the ES of the sample with 1.6 mm ILD increased by 7.5% after 10 load cycles at an initial nominal displacement of 0.2 mm (Figure 19, left), while in the case of an initial nominal displacement of 0.4 mm (Figure 21, left), the increase was 10.2%. Doing the same to samples with 2.4 mm ILD yielded an increase in the ES of the sample of 8.4% and 9.8%, respectively. In Table 4, the changes in the ES of the sample are summarized. It is evident that by loading the samples after printing, the sample stiffness increased and the ES of the sample kept increasing slowly after a number of cycles.

## 4. Conclusions

In this study, two types of samples with different ILD were produced by FFF technology from PLA material by an Ultimaker 3 3D printer using the same technological parameters.

The effects that the orientation of the samples and the movement path of the nozzle have on the shape of the samples were also studied. By selecting and examining the samples with an ILD of 1.6 mm and 2.4 mm, it was determined that the shape quality of the infill lines was related to the movement path of the nozzle.

The samples with two different ILD of 1.6 mm and 2.4 mm were subjected to loading–unloading cycles and the effect of such cyclic loading on the mechanical properties was investigated. Each sample was subjected to 10 cycles of compressive loading to a certain amount of displacement. Force–displacement curves were drawn for samples with 1.6 mm and 2.4 mm ILD. Comparing the curves, it can be seen that the curve of the first cycle was significantly different from that of the subsequent cycles. Hysteresis rings showed that the samples hardened under cyclic loading. Testing and analyzing the effect of cyclic loading on the ES of the sample were also performed for two different modes. In the first case, cyclic loading and unloading was performed from the displacement value of 0.2 mm to 0.5 mm with a step of 0.1 mm. In the second case, the sample was loaded from 0.4 mm to 0.5 mm. In both cases, 10 cycles were performed for each step.

It was observed that the compressive cycling loading increased the ES of the sample with the ILD 1.6 mm for 7.5% after the first 10 cycles at an initial nominal displacement of 0.2 mm, where the largest change was observed after the second loading cycle. A similar effect with samples with a 2.4 mm ILD showed an 8.4% increase in the ES of the sample. This effect was even more distinct in the case of cycling loading from an initial nominal displacement of 0.4 mm, where the increase in the ES of the sample was 10.2% and 9.8% for an ILD of 1.6 mm and 2.4 mm, respectively. The largest increase in the ES of the sample for 11% was observed after 20 loading cycles from an initial nominal displacement of 0.4 mm at 1.6 mm ILD.

## Figures and Tables

**Table 1:** An overview of printing speeds, printing temperatures, and layer heights used in different papers.

	No. of Article	Printing Speed [mm/s]	Printing Temperature [°C]	Layer Height [mm]
PLA	Chacón [19]	20, 50, 80	210 °C	0.12, 0.18, 0.24
PLA	Mercado-Colmenero [31]	50	220 ± 10 °C	0.2
PLA	Aloyaydi [34]	20	200 °C	0.2
PLA	Senatov [35]	30	180 °C	0.2

**Table 2:** LVDT displacement transducer.

	Model	Range	Uncertainty	Output
**LVDT Displacement Transducer**	RDP DCTH100AG-1431A-L10	5 mm	1.6 µm	0 V to 10 V

**Table 3:** Parameters of the compression test.

Parameters of Compression Testing	
**Alternating Speed of the Crosshead**	± 1.2 mm/min
**Temperature**	20 °C (room temperature)
**1st Case of Cyclic Loading**	- Ten times to 0.2 mm compression and back until there is no load applied (zero force), - Ten times to 0.3 mm compression and back until there is no load applied (zero force), - Ten times to 0.4 mm compression and back until there is no load applied (zero force), - Ten times to 0.5 mm compression and back until there is no load applied (zero force).
**2nd Case of Cyclic Loading**	- Ten times to 0.4 mm compression and back until there is no load applied (zero force), - Ten times to 0.5 mm compression and back until there is no load applied (zero force).

**Table 4:** Summary of the elastic stiffness (ES) of the sample changes induced by cyclic compressive loading.

	Sample with 1.6 mm ILD						Sample with 2.4 mm ILD					
**Cyclic loading interval**	[0.2 to 0.5] mm			[0.4 to 0.5] mm			[0.2 to 0.5] mm			[0.4 to 0.5] mm		
**No. of load cycles**	1	10	40	1	10	20	1	10	40	1	10	20
**Nominal displacement [mm]**	0.2	0.2	0.5	0.2	0.4	0.5	0.2	0.2	0.5	0.4	0.4	0.5
**Elastic stiffness of the sample [MPa]**	2889	3105	3178	2836	3125	3147	2803	3039	3077	2795	3069	3080
**Elastic stiffness of the sample increase [%]**	/	7.5	10.0	/	10.2	11.0	/	8.4	9.8	/	9.8	10.2
